# The Salivary Tumor That Lost Its Way: A Case Report of Sinonasal Pleomorphic Adenoma

**DOI:** 10.7759/cureus.104061

**Published:** 2026-02-22

**Authors:** Áine O'Brien, Eoin F Cleere, Thomas Crotty, Mohammad Habibullah Khan

**Affiliations:** 1 Department of Otolaryngology, South Infirmary Victoria University Hospital, Cork, IRL; 2 Department of Otolaryngology - Head and Neck Surgery, South Infirmary Victoria University Hospital, Cork, IRL; 3 Department of Otorhinolaryngology, South Infirmary Victoria University Hospital, Cork, IRL

**Keywords:** nasal obstruction, nasal obstruction surgery, pleomorphic adenoma, sinonasal pathology, unilateral nasal obstruction

## Abstract

Pleomorphic adenoma (PA) is a benign tumour that most commonly arises in the salivary glands but can occur in other sites. Occurrence in the nasal cavity is rare, arising from minor salivary tissue. A 46-year-old man with primary ciliary dyskinesia and type 2 diabetes presented with bilateral nasal obstruction and chronic rhinosinusitis. Examination showed bilateral nasal polyposis, with the right one being more obstructive. Computed tomography (CT) demonstrated bilateral diffuse pansinus mucosal thickening with polypoidal transformation. The patient underwent bilateral endoscopic sinus surgery. Nasal polyps were sent for a routine histological examination. Histology revealed sheet-like fascicular growth of uniform ovoid-spindled cells with biphasic ductal epithelial-like and myoepithelial architecture, in keeping with PA. Targeted RNA sequencing detected an NCALD::PLAG1 fusion, supporting a salivary cell origin, with PLAG1 alterations characteristic of PA. PA of the nasal cavity, although rare, should be considered in the differential diagnosis of unilateral nasal obstruction or mass. Surgical excision remains the definitive treatment. Long-term clinical surveillance is advised, considering the risk of later recurrence or malignant transformation.

## Introduction

Pleomorphic adenoma (PA) is the most common benign salivary gland neoplasm, accounting for approximately 75% of all salivary gland tumours [1]. It most frequently arises in the parotid gland (approximately 84%), followed by the submandibular gland (8%) and minor salivary glands (6-7%) [2]. These tumours typically present between the third and sixth decades of life and demonstrate a female predominance. Clinically, they manifest as slow-growing, painless, well-circumscribed masses. Diagnosis may be suspected by radiological or cytological investigation and is confirmed on histopathological examination [3].

Although PA predominantly arises within the major salivary glands, it may also develop in ectopic or minor salivary gland sites, including the soft palate, larynx, epiglottis, and sinonasal tract [4]. Sinonasal PA is rare and poses unique diagnostic challenges. In contrast to their major salivary gland counterparts, sinonasal tumours frequently exhibit hypercellularity and a relative paucity of myxoid stroma, potentially leading to misdiagnosis as a malignant neoplasm. Furthermore, while PAs are benign, incomplete excision carries a risk of recurrence and malignant transformation to carcinoma ex-PA [5].

We report a case of hypercellular sinonasal PA arising in the setting of chronic rhinosinusitis, with molecular confirmation of an uncommon NCALD::PLAG1 gene fusion, highlighting the diagnostic challenges and the value of adjunct molecular testing.

## Case presentation

A 46-year-old man with a background of primary ciliary dyskinesia, situs inversus with dextrocardia, and type 2 diabetes mellitus presented to the otolaryngology outpatient clinic. He presented with progressive right-sided nasal obstruction and longstanding symptoms of chronic rhinosinusitis, including nasal congestion and facial pressure, ongoing for three years. There was no history of significant epistaxis, weight loss, or facial numbness.

**Figure 1:**
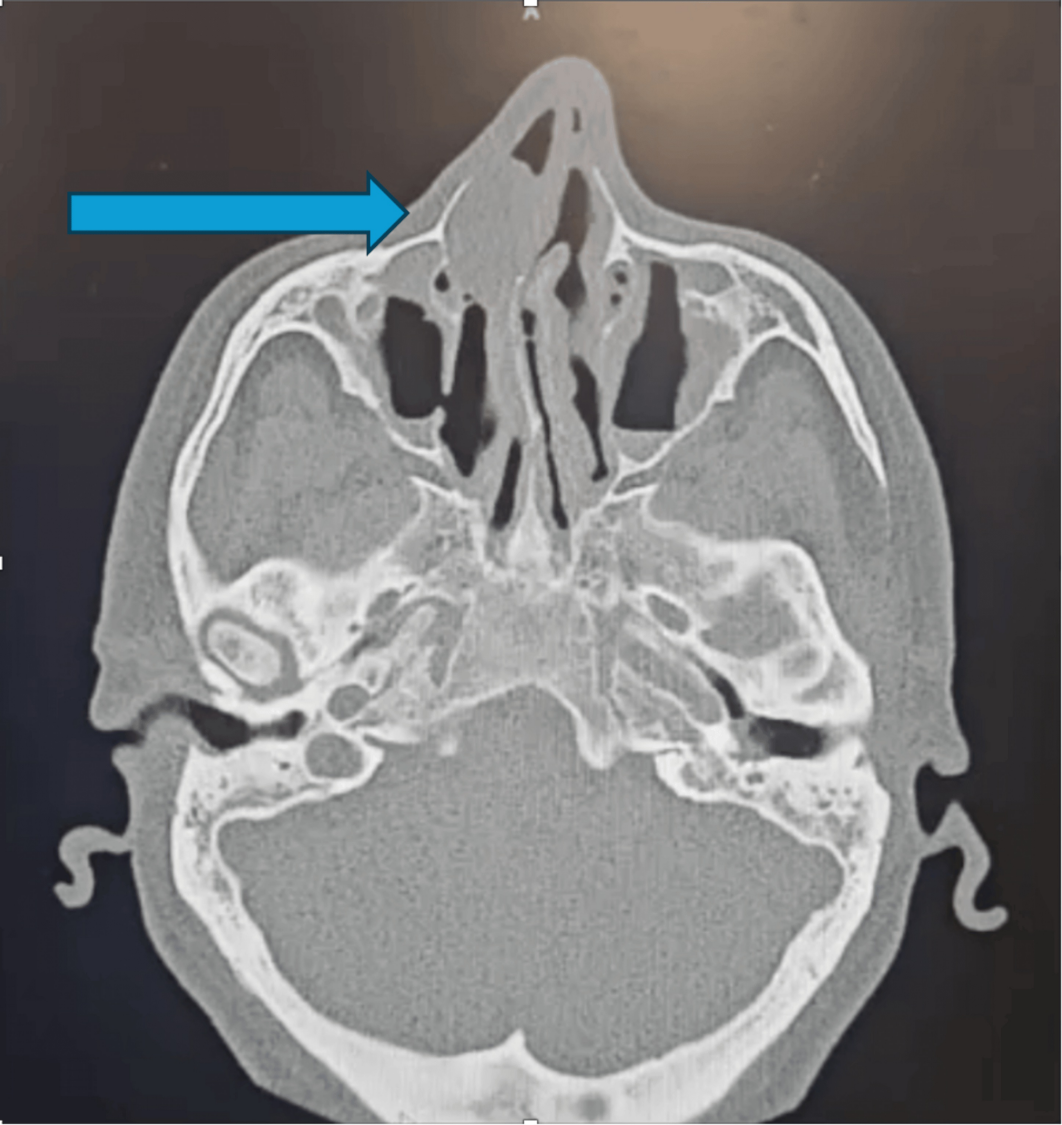
CT sinuses: dominant right-sided nasal cavity lesion causing obstruction on a background ofbilateral diffuse mucosal thickening with polypoid changes

**Figure 2:**
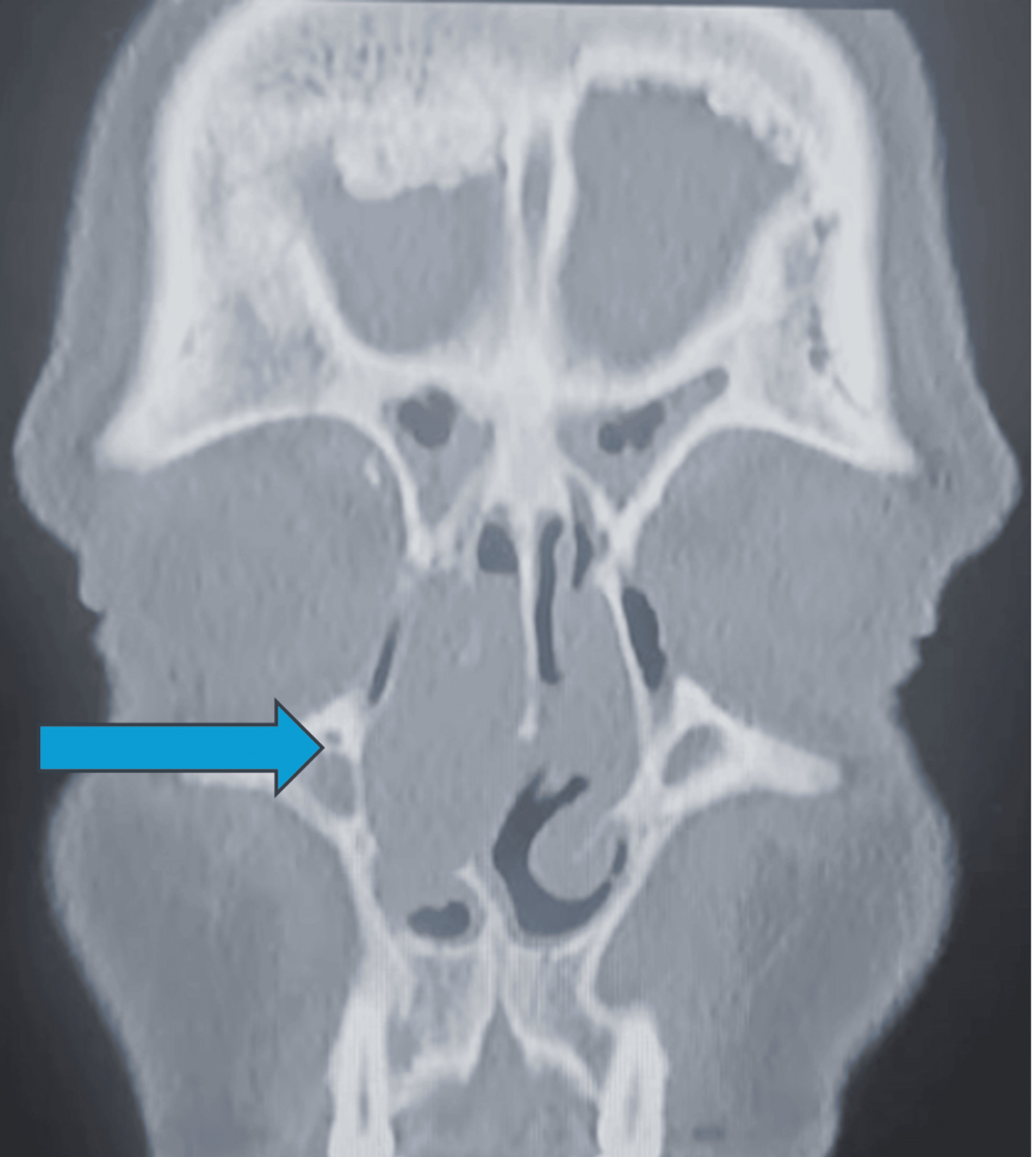
CT sinuses: dominant right-sided nasal cavity lesion causing obstruction on a background of bilateral diffuse mucosal thickening with polypoid changes

Flexible nasoendoscopy demonstrated a right-sided obstructive nasal polypoidal mass with diffusely inflamed and erythematous mucosa bilaterally. The lesion appeared polypoid and was clinically suspected to represent an inflammatory nasal polyp in the context of chronic rhinosinusitis. Computed tomography (CT) of the paranasal sinuses revealed bilateral diffuse mucosal thickening with polypoid changes and a dominant right-sided nasal cavity lesion causing obstruction (Figures 1, 2). There was no evidence of bony erosion or destructive change. Given the patient’s symptom burden, unilateral obstructive mass, and radiologic findings, he underwent endoscopic sinus surgery and excision of the mass. The mass was grossly excised in its entirety endoscopically and submitted for histopathological evaluation. Intraoperatively, the lesion appeared similar to an inflammatory polyp, without necrosis, ulceration, or obvious invasion. Other differentials included an anterochoanal polyp and an inverted papilloma. 

Histological examination of the right nostril polyp demonstrated a cellular proliferation with sheet-like and focally fascicular architecture, composed of uniform ovoid to spindled cells within a fibromyxoid stroma and focal adipocytic metaplasia. No high-grade features, tumour necrosis, or lymphovascular invasion were identified.

Immunohistochemical studies were performed to confirm the suspected diagnosis, considering the unusual site of the suspected pathology. Immunohistochemistry showed diffuse MNF116 and SOX10 positivity, with AE1/AE3 and CK7 highlighting epithelial elements, and S100 and SMA confirming myoepithelial differentiation. The tumour cells were negative for beta-catenin (wild-type), INSM1, synaptophysin, TTF-1, CD31, CD34, STAT6, and desmin. These findings supported a diagnosis of PA; however, given the relative stromal paucity and diagnostic uncertainty, molecular testing was pursued.

Targeted RNA sequencing identified an NCALD::PLAG1 gene fusion. Rearrangements involving PLAG1 are well-recognised oncogenic drivers in PA and provided molecular confirmation of the diagnosis [6]. Due to the discordant findings, the specimen was also sent to a world-leading expert on salivary tumours, who concurred with the diagnosis of PA. Postoperatively, no tumour was evident on clinical examination. There is no evidence for follow-up imaging to monitor recurrence of PA when the initial lesion was excised in its entirety. At the most recent follow-up, 12 months post-surgery, the patient remained well with no evidence of recurrence. He will remain under close clinical surveillance for at least six years, as this is the median length of time of PA recurrence. 

## Discussion

PA of the sinonasal tract is rare, with most cases arising from minor salivary glands of the nasal septum. Less commonly, lesions originate from the lateral nasal wall or paranasal sinuses. Patients typically present with unilateral nasal obstruction, congestion, epistaxis, hyposmia, or facial pressure [7]. Because these symptoms overlap significantly with inflammatory sinonasal disease, lesions may initially be presumed to represent nasal polyps, as in this case.

Histologically, PA is characterised by three components: epithelial ductal structures, myoepithelial cells, and a mesenchymal-like stroma that may be myxoid, chondroid, or myxochondroid [8]. However, sinonasal PA frequently demonstrates reduced stromal elements and increased cellularity compared with tumours of the major salivary glands [9]. This hypercellular pattern can mimic other spindle cell or epithelial neoplasms of the sinonasal tract, including adenoid cystic carcinoma, myoepithelioma, basal cell adenoma, and low-grade adenocarcinoma [10]. Recognition of the biphasic architecture and confirmation with immunohistochemistry are therefore critical. Molecular alterations provide additional diagnostic support. PLAG1 (pleomorphic adenoma gene 1) is a proto-oncogene encoding a zinc finger transcription factor. In PA, chromosomal rearrangements involving PLAG1 lead to promoter swapping and overexpression.

PLAG1 fusions are among the most common molecular alterations in PA across anatomical sites [6]. Numerous fusion partners have been described; however, NCALD is an uncommon partner, and the true prevalence of this rearrangement remains uncertain, as molecular testing is not routinely performed in all cases. In diagnostically challenging lesions, in particular those with unusual morphology, molecular analysis can provide valuable confirmation to ensure adequate treatment and clinical follow-up. 

Complete surgical excision with negative margins remains the definitive management for sinonasal PA. Enucleation or incomplete excision is associated with recurrence. Although PA is benign, there is a recognised risk of malignant transformation to carcinoma ex-PA, estimated at approximately 1.5% within five years and increasing with prolonged duration [3]. Data specific to sinonasal lesions are limited, but PA is associated with a potential risk of recurrence, with reported mean or median times to recurrence of approximately 6 years, and recurrences documented even decades after initial excision [11, 12]. In the sinonasal tract, complete excision with wide margins may be challenging to achieve endoscopically due to anatomical constraints. Identification of this entity on histopathology may prompt consideration of further formal resection in selected cases and, at a minimum, long-term clinical surveillance compared to nasal polyps, which do not require long-term follow-up. Accurate pathological diagnosis is therefore critical in guiding appropriate follow-up to detect potential recurrences or malignant transformations.

## Conclusions

Sinonasal PA is a rare but important differential diagnosis in patients presenting with unilateral nasal obstruction. Hypercellularity and stromal paucity may create significant diagnostic uncertainty and mimic malignant neoplasms. Recognition of characteristic biphasic morphology, supported by immunohistochemistry and molecular confirmation of PLAG1 rearrangements, was essential for accurate diagnosis. Complete surgical excision with long-term follow-up is recommended to detect recurrence early and monitor for malignant transformation.
